# Effects of a therapeutic lifestyle change diet and supplementation with Q10 plus L-carnitine on quality of life in patients with myocardial infarction: A randomized clinical trial

**DOI:** 10.15171/jcvtr.2017.03

**Published:** 2017-03-06

**Authors:** Mohammad Hossein Sharifi, Mohammad Hassan Eftekhari, Mohammad Ali Ostovan, Abbas Rezaianazadeh

**Affiliations:** ^1^School of Nutrition and Food Sciences, Shiraz University of Medical Sciences, Shiraz, Iran; ^2^Department of Cardiology, School of Medicine, Shiraz University of Medical Sciences, Shiraz, Iran; ^3^Colorectal Research Center, Shiraz University of Medical Sciences, Shiraz, Iran

**Keywords:** Diet Therapy, Coenzyme Q10, Carnitine, Quality of Life, Myocardial Infarction

## Abstract

***Introduction:*** Myocardial infarction (MI) has a deleterious effect on quality of life (QoL), which can affect cardiac prognosis after MI. Thus, new strategies have to be identified for improving the QoL. To our knowledge, no studies have been conducted on the impact of therapeutic lifestyle change (TLC) diet and L-carnitine plus Q10 supplementation on QoL after MI.

***Methods:*** The study aimed to measure 128 MI patients’ QoL using MacNew QoL questionnaire (global scales and physical, emotional, and social subscales) before and 3 months after the intervention. The patients were divided into 4 groups. Group A received TLC diet, group B orally received Q10 150 mg/d and L-carnitine 1200 mg/d, and group C received a combination of carnitine plus Q10 and TLC diet. Finally, group D, as the control group, only underwent the routine care.

***Results:*** The results showed a significant increase in MacNew questionnaire’s physical, emotional, and social subscales in the four groups after the intervention. The results of within-group analysis showed that the physical and emotional subscales changed significantly (*P* < 0.001 and *P* < 0.022, respectively). In the emotional subscale, TLC group showed a significant improvement compared to groups B and D (*P* < 0.019 and *P* < 0.001, respectively), but not group C (*P* < 0.681). In the physical subscale, Q10 plus L-carnitine group showed a significant improvement compared to groups A and D (*P* < 0.001 and *P* < 0.0001, respectively), but not group C (*P* < 0.860). In the global scale, combination of carnitine plus Q10 and TLC diet group demonstrated a considerable improvement compared to groups A, B, and D (*P* < 0.001, *P* < 0.001, and *P* < 0.001, respectively). Nevertheless, the results of within-group analysis revealed no significant differences among the four groups regarding the social subscale (*P* < 0.229).

***Conclusion:*** Both TLC diet and supplementation with Q10 and L- carnitine had a positive effect on the physical and emotional subscales of MacNew questionnaire and may improve post-MI prognosis. Based on the results, combination of Q10 plus L-carnitine and TLC die can be a potential intervention for improving QoL and secondary prevention.

## Introduction

Myocardial infarction (Ml) has a deleterious effect on quality of life^1^ (QoL), which can impact cardiac prognosis after MI.^2^ QoL is a strong, independent predictor of health outcomes, including mortality and cardiovascular events.^3^ It has also shown that low QoL was the potent predictor of rehospitalization during a 3-year period after percutaneous coronary intervention (PCI).^4,5^ Moreover, a study by Wang et al revealed that QoL could determine the priority and necessity of post MI interventions.^2^ Since cardiovascular disease remains a common cause of death, experts have emphasized on utilization of fundamental strategies to decrease cardiovascular risk factors and improve QoL through changing lifestyle and diet.^6-8^

Evidence has shown that most patients with MI have cardiovascular risk factors related to lifestyle and diet.^9^ Besides, lifestyle intervention programs could improve QoL in post MI patients.^10^ There are a variety of lifestyle modification programs and recommendations regarding diet and rehabilitation programs based on patients’ individual characteristics. Patients who had previously undergone coronary artery bypass graft (CABG) surgery responded to an intense lifestyle intervention program with significantly improved QoL.^11^ Additionally, a previous study indicated that the trans fat intake^12^ or Mediterranean diet^13^ could affect the QoL. Also, therapeutic lifestyle change (TLC) diet intervention, as a lifestyle modification program, effectively modified cardiac risk factors in post-operative CABG.^11^ Therefore, incorporating a TLC diet intervention into PCI and MI postoperative programs might modify the cardiac risk factors, need for further PCI procedures,^14^ and improve the QoL.

It is shown that total antioxidants intake has a relationship with improvement in QoL.^15^ Previous studies have reported L-carnitine^16^ and Q10^15-17^ as potent antioxidants, each having a positive effect on QoL. L-carnitine plays an important role in energy production in the myocardium and inhibits fatty acid ester reposition that occurs during ischemic events.^18^ In addition, L-carnitine supplementation had positive impacts on muscle cells and red blood cells in hemodialysis patients, which might improve the overall sense of well-being and enhance the QoL.^16^ Previous studies also revealed that multi-nutrient supplements, including Q10, improved the myocardial function, ejection fraction and QoL.^15^ Although currently one promising therapy for improving cardiac health is the use of dietary supplementations, much uncertainty still exists about the impact of dietary supplementation with L-carnitine and Q10 on QoL.

To our knowledge, no studies have yet been conducted on the effect of TLC diet and its combination with dietary supplement with L-carnitine plus Q10 on QoL after MI. The present study aims to determine the effects of TLC diet and supplement therapy with Q10 and L-carnitine on QoL after MI.

## Materials and methods

### Design and sample size

This was a single-blind randomized controlled trial with 2*2 factorial design. Based on mean differences in QoL, type I error of 5%, power of 80%, and 20% probability of loss, a 128-subject sample size was determined for the study (32 subjects in each group).

### Inclusion and exclusion criteria

Acute myocardial infarction (AMI) was diagnosed based on the European Society of Cardiology/American College of Cardiology (ESC/ACC) diagnostic criteria for AMI. The inclusion criteria of the study were the first experience of STMI, aging 40-65 years, being candidate for primary PCI within 12‏ hours after MI, low density lipoprotein (LDL) > 130 mg/dL (measured within 24 hours after Ml), left ventricle ejection fraction ≥ 30, receiving atorvastatin 80 mg daily, having no history of cardiac events after successful PCI, arrhythmia, and congestive heart failure.

The exclusion criteria were use of warfarin and antioxidants such as vitamin C, E, and omega 3, lipid lowering diet in the previous 6 months, presence of documented psychological diseases and withdrawal from the study before 60 days after the intervention.

### Sampling and random assignment

**Figure 1:**
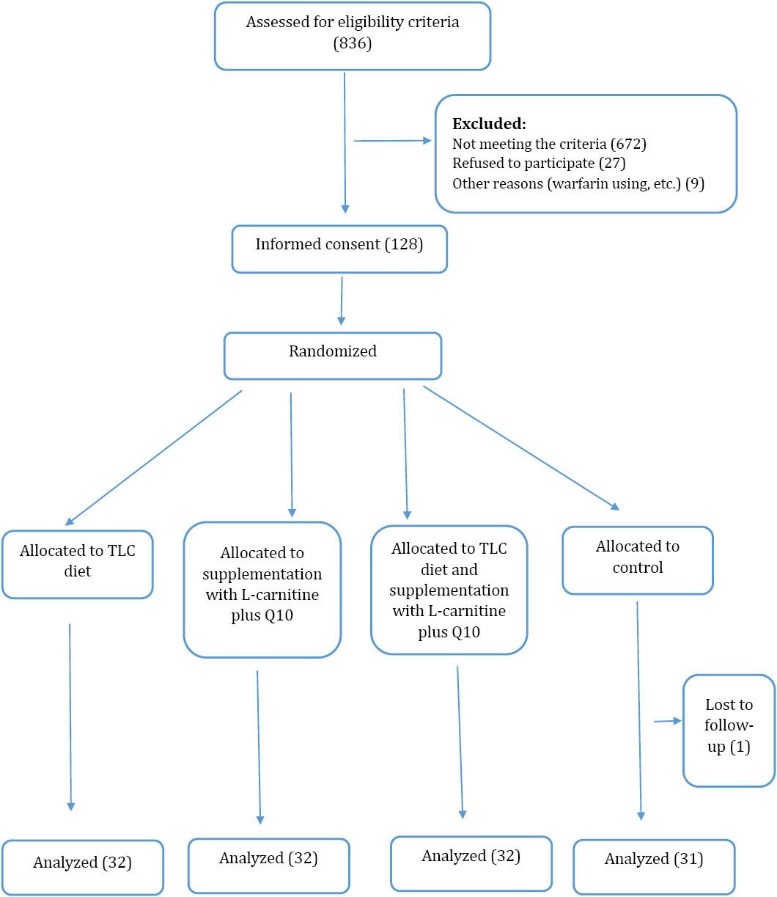
Consort flow diagram of participants’ recruitment in the study.

At first, the research population included 836 MI patients who were admitted in Al-Zahra and Kowsar heart hospitals. According to the inclusion and exclusion criteria of the study, 128 patients were selected, provided with a full explanation about the study objectives, and signed the written informed consent forms (Figure 1). Then, a 2-step simple randomization was used. Accordingly, once the participants were randomized for each treatment and then, they were divided into the study groups through block randomization.

### Intervention

The patients were divided into 4 groups. The patients in the TCL diet group (group A) received TCL diet in accordance with their weight and individual characteristics. The patients in the supplement group (group B) received two supplements; L-carnitine 1200 mg (produced by Karen Company, Iran) and Q10 150 mg (produced by LYNAE Company, USA). The patients in the TLC diet and supplement group (group C) received TLC diet and 2 supplements, namely L-carnitine 1200 mg and Q10 150 mg. Finally, the patients in the control group (group D) only received the routine care. All the patients were followed for 3 months.

### The TLC diet planning

First, a 3-day food intake record was used to evaluate the patients’ diets at baseline and at the end of the intervention. The food intake records data were converted to energy and nutrients data and were analyzed using the NUT 4 software. Then, a 7-day TLC diet plan consisting of 3 meals and 3 snacks was designed, which had to be continued for 90 days. In addition, the patients were educated for 30 minutes and were provided with an educational brochure. Accordingly, the TLC program (11, 19) included limiting the intake of saturated fatty acids (SFA) (<7% of total daily calories), cholesterol (<200 mg/d), monounsaturated fatty acids (MUFA) (10%-20%), and polyunsaturated fatty acids (PUFA) (<10%), increasing phytosterols (comprising plant sterols and stanols) (two servings per day) and water soluble fiber (10–25 g/d), having moderate intensity physical activity for 20 minutes at least 3 times a week calculated by Physical Activity Questionnaires (IPAQ), and cessation of smoking . The participants were visited after 45 and 90 days. It should be noted that the participants could contact the researcher through text massages if they had any concerns during the study period.

### Measurements of physical activity

IPAQ is an instrument designed primarily for adults^20^ (age range of 15-69 years). The IPAQ face-to-face interview format was used to assess the habitual physical activity during the previous 7 days before MI. Additionally, energy consumption was calculated based on the second edition of codes and metabolic equivalent (MET) values. The IPAQ data were converted to MET scores (MET- min/wk) for each type of activity by multiplying the number of minutes dedicated to each activity class by the specific MET score for that activity. Moreover, based on the revised scoring protocol 2005,^21^ physical activity levels were categorized into 3 levels as follows: high (at least 3000 MET-min/wk), moderate (at least 600 MET-min/wk, and low (less than 600 MET-min/wk). Sitting time^20^ is yet a new important variable that can be extracted from this questionnaire. This additional indicator variable is defined as the time spent in a sedentary position and is not included as a score of physical activity. This variable was calculated based on minute per day.

### MacNew quality of life questionnaire

The QoL data were obtained using MacNew questionnaire,^22^ which is a self-administered questionnaire for assessment of QoL among MI patients using a 2-week time frame. This questionnaire is superior to SF-36 questionnaire for assessing QoL.^23^ International MacNew QoL questionnaire has also been reported to be a valid and reliable instrument in Iran.^24^ This questionnaire is responsive and sensitive to changes in QoL following various interventions for patients with heart diseases. MacNew questionnaire consists of 27 questions about symptoms, such as angina/chest pain, shortness of breath, fatigue, dizziness, and aching legs. These questions are categorized into three sections, namely social function (13 items), physical function (13 items), and emotional function (14 items). Each item is scored using a 7-point Likert scale ranging from 1 (low QoL) to 7 (high QoL), with the minimal important difference of 0.50 points.

### Statistical analysis

Continuous variables were expressed as mean ± standard deviation (SD), while categorical ones were presented as absolute numbers and percentages. First, normal distribution of the data was evaluated through Kolmogorov-Smirnov test. Then, Wilcoxon and Kruskal–Wallis tests were used for comparison of the four study groups, and Mann–Whitney U test with Bonferroni correction was used for post hoc comparisons. In addition, differences among the four groups were assessed using mean difference with standard error of mean (SEM). Besides, MacNew questionnaire’s global and 3 subscales scores before and after the intervention were expressed as median ± the first and third quartiles. Paired *t* test was used for assessment of dietary intake indices before and after intervention. Analysis of variance (ANOVA) was also employed to analyze the effects of the dietary intervention. The relationships between different variables were examined using Pearson’s correlation coefficient. All statistical analyses were performed using the SPSS 19 and significance level was set at 0.05. Graph Pad Prism was also used to draw the graphs.

## Results

This study evaluated the effects of three interventions on MacNew questionnaire’s global and subscales scores. Among the 836 patients screened for eligibility, 128 were enrolled into the study. The patients’ characteristics have been presented in Table 1. The results revealed no significant differences among the study groups regarding age, sex, education level, and work status, ejection fraction, and comorbidities, number of involved vessels in PCI, TLC diet characteristics, and physical activity levels. Among the patients, 91% were male and 93% were married. In addition, the most frequently reported comorbidities were hypertension (34.3%), diabetes (27.3%), smoking (21.8%), obesity (6.2%), single vessel disease (64.5%), two-vessel disease (24.5%), and multi-vessel disease (11%). Also, 90.6% of the participants had ST-segment elevation MI (STMI).

**Table 1:** The patients’ socio-demographic and clinical characteristics

	**Group 1** ** (n = 32)**	**Group 2** ** (n = 32)**	**Group 3** ** (n = 32)**	**Group 4** ** (n = 31)**	***P*** ** value**
Age year (mean ± SD)	50± 10	54± 8	51± 9	51± 10	0.28
Gender (male) (%) (number)	87.5 (28)	96.8 (31)	87.5 (28)	81.2 (26)	0.16
Marital status (%) (number)					
Married	93.7 (30)	90.6 (29)	87.5 (28)	87.5 (28)	0.18
Single	6.2 (2)	9.3 (3)	9.3 (3)	6.2 (2)	
Widowed	0	0	3.1 (1)	3.1 (1)	
Education status (%) (number)					
Below high school	56.2 (18)	50.0 (16)	46.8 (15)	50 (16)	0.18
High school	25.0 (8)	31.2 (10)	28.1 (9)	25 (8)	
Academic	18.7 (6)	18.6 (6)	25.0 (8)	21.8 (7)	
Work‏ status (%) (number)					
Working	53 (17)	56.2 (18)	46.8 (15)	43 (14)	0.32
Not working	25.0 (8)	28.1 (9)	25.0 (8)	21.8 (7)	
Retired	21.8 (7)	18.6 (6)	28.1 (9)	31.2 (10)	
Satisfied with financial status (%) (number)	78.1 (25)	84.3 (27)	75.0 (24)	81.2 (26)	0.22
One-vessel disease (%) (number)	62.5 (20)	65.6 (21)	62.5 (20)	65.6 (21)	0.98
Two-vessel disease (%) (number)	25.0 (8)	28.1 (9)	21.8 (7)	21.8 (7)	
Multi-vessel disease (%) (number)	12.5 (4)	6.2 (2)	15.6 (5)	9.3 (3)	
Hypertensive History (%) (number)	34.3 (11)	37.5 (12)	31.2 (10)	34.3 (11)	0.94
Diabetic History (%) (number)	28.1 (9)	31.2 (10)	21.8 (7)	28.1 (9)	0.98
Smoker (%) (number)	21.8 (7)	31.2 (10)	18.7 (6)	28.1 (9)	0.88
BMI (kg/m^2^) >30, (%) (number)	9.3 (3)	6.2 (2)	3.1 (1)	6.2 (2)	0.19
Ejection fraction (mean ± SD)	45±9.3	42±8.7	46 ±9.3	46 ±7.6	0.13
STMI (%) (number)	90.6 (29)	87.5 (28)	90.6 (29)	93.7 (30)	0.20

Abbreviations: BMI, body mass index; STMI, ST elevation myocardial infarction.

According to Table 2, no significant difference was found among the four study groups regarding dietary intake at baseline. The results indicated that the participants recruited in groups A (TLC diet) and C (TLC diet and supplementation with LC plus Q10) followed the instructions given by the research team and fulfilled TLC diet plan. The result also showed no significant changes in dietary intake in groups B (supplementation with LC plus Q10) and D (control).

**Table 2:** The participants’ dietary intake and physical activity before and after the intervention

**Parameter**	**Groups**												***P*** *
	**A (n=32)**			**B (n=32)**			**C (n=32)**			**D (n=32)**			
	**Before**	**After**	***P*** ^#^	**Before**	**After**	***P*** ^#^	**Before**	**After**	***P*** ^#^	**Before**	**After**	***P*** ^#^	
SFAs (%)	13	7	.04	14	12.5	.14	13	8	.01	14	13	.23	.0001
MUFAs (%)	7.5	12.8	.02	8	8.3	.23	7.6	13	.003	7.2	8.1	.35	.0001
PUFAs (%)	14.5	9.5	.003	15	14	.67	14	10	.042	13.6	14	.44	.0001
Cholesterol(mg/dL)	238±11	162±19	.0001	243±16	238±19	.055	247±18	160±26	.0001	236±14	231±19	.07	.0001
Water soluble fiber intake (g/d)	7±2.7	18±3.1	.0001	7±2.2	7±1.8	.286	8±3.0	18±4.0	.0001	7±.2.6	7±2.4	.44	.0001
Physical activity (MET/minute)	368±73	518±84	.0001	367±67	388±81	.108	396±78	573±101	.0001	368±72	388±89	.11	.0001

^#^Paired *t* test p-values calculated based on mg/d for SFAs, MUFAs, and PUFAs; *ANOVA *P* value after the intervention. Group A, TLC diet; Group B, supplementation with Q10 plus L-carnitine; Group C, supplementation with Q10 plus L-carnitine and TLC diet; Group D, control. SFA, saturated fatty acids; MUFAs, monounsaturated fatty acids; PUFAs, polyunsaturated fatty acids.

IPAQ was assessed at baseline and at the end of the intervention. The results showed that 78%, 22%, and 0% of the participants in group A had low, moderate, and high physical activity level, respectively. After the intervention, however, these values changed to 37%, 67%, and 0%, respectively. In group C, 74%, 26%, and 0% of the participants had low, moderate, and high physical activity levels, respectively at baseline. After the intervention, however, these measures changed to 40%, 60%, and 0%, respectively. Interestingly, the results showed no significant differences between the two groups at baseline and at the end of the intervention (*P* < 0.07).

Moreover, the means of baseline sitting times were 355 and 364 minutes in groups A and C, respectively, which respectively changed to 256 and 244 MET per minute at the end of the intervention (*P* < 0.0001, *P* < 0.0001).

Evaluation of the subjects’ smoking behavior indicated that smoking cessation after the intervention dropped dramatically to 5% in the four groups. Indeed, no significant difference was found among the 4 groups in this regard (*P* < 0.635).

The 4 groups were also compared with respect to MacNew questionnaire’s global and three subscales scores and the results have been presented in Table 3. Accordingly, MacNew questionnaire’s global and three subscales scores improved significantly after the intervention. Nevertheless, the results of post hoc analysis revealed no significant differences among the 4 groups regarding the social subscale (*P* < 0.229).

**Table 3:** MacNew global and three subscales scores before and after the intervention

**Parameter**	**Groups**												***P*** *
	**A (n = 32)**			**B (n = 32)**			**C (n = 32)**			**D (n = 32)**			
	**Before**	**After**	***P*** ^#^	**Before**	**After**	***P*** ^#^	**Before**	**After**	***P*** ^#^	**Before**	**After**	***P*** ^#^	
Global	4.5(4.0-5.0)^acd^	5.0( 5.0-5.5 )	.0001	4.8(4.4 -5.1)	5.3(5.0-5.6)^bcd^	.0001	4.7(4.5-5.0)	5.5(5.3-5.6)^cd^	.0001	4.7(4.4-4.9)	5.0(4.9-5.3 )	.0001	.0001
Physical	4.5(4.0 -5.0)	5.0( 5.0-5.5)^abcd^	.0001	4.5(4.0 -5.0)	5.5(5.0-6.0 )^bd^	.0001	4.5(4.0-5.0)	5.0(5.0-5.5)^cd^	.0001	4.5(4.0-5.0)	5.0(4.5-5.4 )	.0001	.0001
Emotional	4.6( 4.1 - 4.8)	5.2(5.0-5.5)^ad^	.0001	4.6(4.3- 4.8)	5.0( 4.5 - 5.2)	.0001	4.6(4.3-4.8)	5.2(4.9-5.7)^cd^	.0001	4.6(4.1-4.8)	5.2(5.0-5.5)	.0001	.022
Social	4.7( 4.7 - 5.6)	4.5(5.3-5.8)	.0001	5.2(4.7 - 5.6)	5.5(5.0-6.02)	.0001	4.7(4.2-5.1)	5.3(5.0-5.8)	.0001	5.0(4.5-5.2)	5.5(5.1-5.8)	.0001	.229

^#^Wilcoxon signed ranks *P* value, *Kruskal–Wallis between-group *P* values after the intervention. Data expressed as median ± the 1st and 3rd quartiles. P <0.05 in between groups (groups A, B, C and D presented with a, b, c and d). Group A: TLC diet; Group B: supplementation with Q10 plus L-carnitine; Group C: supplementation with Q10 plus L-carnitine and TLC diet; Group D: control.

**Figure 2:**
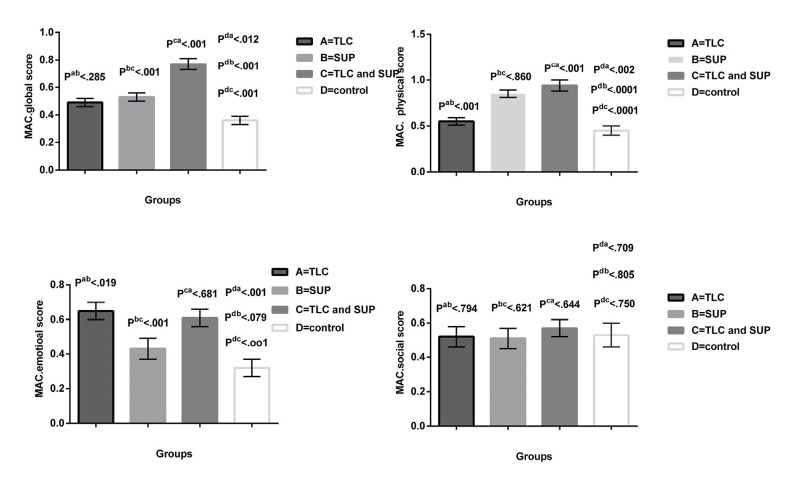
Mean difference and SEM of MacNew questionnaire’s global, physical, emotional, and social scores. P values in between groups ( groups A, B, C, D presented with a, b, c, and d).

Comparison of the mean differences and SEM of MacNew questionnaire’s global and 3 subscales scores has been shown in Figure 2. As the Figure 2 depicts, the mean difference of the physical subscale improved significantly in groups A, B, and C compared to the control group (*P* < 0.002, *P* < 0.0001, *P* < 0.0001, respectively). Besides, the interventions used in groups B and C had a significant effect on improvement of the physical subscale compared to group A (*P* < 0.001, *P* < 0.001, respectively). However, no significant difference was observed between the interventions utilized in groups B and C with regard to improvement of the physical subscale (*P* < 0.860).

According to Figure 2, the emotional subscale scores significantly improved in groups A, and C compared to the control group (*P* < 0.001, *P* < 0.001, respectively). Besides, the interventions used in groups A and C had a significant impact on this subscale compared to group B (*P* < 0.019, *P* < 0.001, respectively). Also, no significant difference was observed between these two interventions regarding improvement of QoL (*P* < 0.681).

The results indicated no significant differences between groups A, B, and C and the control group concerning the mean difference of the social subscale scores (*P* < 0.709 *P* < 0.805, *P* < 0.750, respectively). Thus, all interventions had similar effects on improving the score of the social subscale of MacNew questionnaire.

Comparison of the changes in the mean difference of MacNew questionnaire’s global and subscales scores showed that the intervention used in group C had a significant effect on increasing QoL compared to groups A, B, and D (*P* < 0.001, *P* < 0.001, and *P* < 0.001, respectively). Although groups A and B were significantly different from the control group in this respect (*P* < 0.012 and *P* < 0.001, respectively), there were no significant differences between groups A and B (*P* < 0.285).

The study results demonstrated no significant correlation between QoL and number of cardiac risk factors (*P* < 0.42, r = 0.3), education level (*P* < 0.40, r = 0.4), and number of involved vessels (*P* < 0.32, r = 0.3).

## Discussion

To our knowledge, this is the first study to investigate the effects of dietary supplementations with Q10 plus L-carnitine and TLC diet on QoL in patients with MI underwent PCI. MI patients usually experience low QoL, which has a negative impact on post-MI outcome. Despite new treatment methods, such as PCI that has a positive effect on QoL, MI still causes major disability in patients. Prior studies have emphasized diet and supplement therapy as adjunct therapies to reduce cardiac risk factors. Thus, identification of the effect of TLC diet and supplement therapy on QoL will probably facilitate the development of new strategies for increasing the QoL and secondary prevention.

Self-reported measures of MacNew questionnaire are frequently used to evaluate the outcome of therapeutic strategies in cardiac patients. This questionnaire is a specific tool for evaluation of QoL in MI patients.^22,24^

The results of the present study demonstrated that TLC diet, dietary supplement with Q10 and L-carnitine, and a mixture of the two interventions significantly improved MacNew questionnaire’s global and physical and emotional subscales scores, while these interventions caused no significant improvement in the score of the social subscale.

The findings also revealed that the effect of supplementation therapy (group B) was similar to both interventions (group C) on improvement of the physical subscale. This might be due to the fact that L-carnitine and Q10 had major effects on increasing the physical subscale. As mentioned before, symptoms of angina or chest pain were included in the physical subscale in MacNew questionnaire. The results of a previous meta-analysis demonstrated that L-carnitine^18^ was associated with a 40% reduction in angina and a 67% reduction in arrhythmia symptoms in patients experiencing AMI. In addition, a previous study indicated that Q10^25^ could relieve statin myalgia, the common drug side effect, that adversely affected all functional and QoL domains.^25^ A case series of 354 patients with statin-associated muscle-related adverse effects also reported muscle pain (93%), fatigue (88%), and weakness (85%).^25^ Therefore, the results of the current study might be attributed to the effect of L-carnitine and Q10 supplementation. To date, no studies have assessed the impact of the combination of L-carnitine and Q10 supplementation on QoL. It has shown that micronutrient supplementation,^15^ including Coenzyme Q10, calcium, magnesium, zinc, copper, selenium, vitamin A, thiamine, riboflavin, vitamin B6, and vitamin B12, could improve QoL scores in elderly patients with heart failure. Intravenous L-carnitine also increased QoL in hemodialysis patients.^16^ Hence, more attention has to be paid to dietary supplementation as an adjuvant therapy for improving QoL. Nonetheless, these results must be interpreted with caution because all patients in this study had received statin.

A previous study demonstrated that the cardiac rehabilitation‏ program had effects on promotion of the physical dimension of QoL based on MacNew questionnaire post MI.^10^ This can be justified by the fact that rehabilitation programs include exercise training, education on heart healthy diets, and counseling to reduce stress. It is notable that TLC diet also consisted of diet plan and exercise, which is similar to rehabilitation‏ programs. Considering the similarities between these 2 interventions, similar outcomes are expected.

The findings of our study indicated a significant improvement in the emotional dimension of QoL in groups A and C that underwent TLC diet intervention. This finding is in agreement with the SUN (Seguimiento Universidad de Navarra) project,^13^ which disclosed that adherence to Mediterranean diet was associated with a better QoL according to SF-36 questionnaire. This results is difficult to explain, but it might be related to the decrease in total fatty acids and saturated fatty acids intake in TLC and Mediterranean diets.^12^ This implies that brain function can influence peripheral signals,^26^ such as amount and type of fatty acids intake. Hence, the participants with higher fat intake might have felt more tired and socially disabled due to emotional problems. However, further research is needed to be undertaken on this topic so as to determine the association between diet intake and QoL more clearly. Another possible explanation for the effect of TLC diet on the emotional subscale is the association between sitting time and mental well-being.^20^ As mentioned before, TLC diet included exercising for 20 minutes at least three times a week. Thus, performing this program was associated with a reduction in sitting time in this study.

The present study findings revealed significant changes in the social dimension scores in the four study groups. This might partially be due to the effect of PCI.^27,28^ Another study indicated that the scores of the physical and social dimensions of MacNew questionnaire increased significantly after PCI.^27^ This can, in turn, lead to improvement in all domains of QoL, especially the social subscale, over time.

Surprisingly, our study results revealed no significant correlations between QoL and number of cardiac risk factors. This does not confirm the findings of other researchers who reported that multiple coronary artery disease risk factors was associated with QoL impairment.^29,30^ The reason for this finding is not clear, but few number of inclusion criteria and limited sample size might have played a role.

## Study strengths and limitations

The major strength of this RCT study was the high response rate obtained from a well-defined trial, (Figure 1). In addition, this study provides data relating to QoL using a MacNew questionnaire,^22^ which is a specific instrument for measurement of QoL. One of the limitations of the present study was that most studies conducted on QoL in Iran have used SF-36 scale, which is a general instrument for measurement of QoL. This caused great difficulty in comprehensive comparison of the results.

## Conclusion

The findings of this study demonstrated that TLC diet and supplement therapy with Q10 and L-carnitine had positive effects on MacNew questionnaire’s global, physical, and emotional subscales scores. Indeed, both interventions had synergistic effects on improvement of MacNew questionnaire’s global score. This study provides an additional treatment option for enhancing QoL, supporting the idea that diet and supplement therapy can affect the QoL. Although the current study was conducted on a small number of participants, the findings suggest that the impact of dietary supplementation on QoL should be confirmed in future studies with larger sample sizes.

## Competing interests

The authors declare that they have no competing interests.

## Ethical approval

The present study was approved by the Ethics Committee of Shiraz University of Medical Sciences. It was also registered in the Iranian Registry of Clinical Trials (IRCT registration number: IRCT2282620150719N1). Written informed consents were obtained from all the participants. Moreover, in case we needed more information about a certain study, we contacted the cor­responding author for obtaining the required information.

## Acknowledgments

This study was financially supported by Shiraz University Medical Sciences (grant No. 93-7457). Hereby, the authors would like to thank the team working on this study and all the participants who cooperated in this trial. They are also grateful for the manager and personnel of Al-Zahra and Kowsar hospitals, Shiraz, Iran. Thanks also go to Ms. Afsaneh Keivanshekouh at the Research Improvement Center of Shiraz University of Medical Sciences for improving the use of English in the manuscript.
